# 小细胞肺癌血清细胞因子的表达及其诊断价值

**DOI:** 10.3779/j.issn.1009-3419.2012.01.03

**Published:** 2012-01-20

**Authors:** 先锋 卢, 雪琴 杨, 宇馨 杨, 咸庆 顾, 玲 廖, 东 王

**Affiliations:** 400042 重庆，第三军医大学大坪医院野战外科研究所肿瘤中心 Cancer Center and Research Institute of Surgery, Daping Hospital, Third Military Medical University, Chongqing 400042, China

**Keywords:** 细胞因子, 肺肿瘤, 诊断, Cytokines, Lung neoplasms, Diagnosis

## Abstract

**背景与目的:**

已有的研究表明：小细胞肺癌（small cell lung cancer, SCLC）患者可出现血清细胞因子表达水平的变化，本研究通过检测SCLC患者血清中细胞因子的差异改变，探讨其在SCLC中的诊断价值。

**方法:**

首先使用Reybiotech G6/G7细胞因子芯片对4例SCLC患者、4例健康人和4例炎症患者血清进行细胞因子差异表达筛查，进一步应用酶联免疫吸附法（ELISA试剂盒）对197例SCLC患者、180例正常对照血清以及97例炎性病变患者血清进行验证。

**结果:**

芯片检测120种细胞因子，从有明显差异的细胞因子中选出最有研究价值的4种进行验证，包括尿激酶型纤溶酶原激活剂受体（urokinase plasminogen activator receptor, uPAR）、人瘦素（Leptin）、巨噬细胞刺激蛋白（macrophage stimulating protein α, MSP-α）和巨噬细胞炎症蛋白1β（macrophage inflammatory protein 1β, MIP-1β）。采用ELISA法验证上述结果，SCLC患者血清uPAR较健康人群以及炎症患者增高（*P* < 0.05），诊断的敏感度为52.93%，特异度为83.36%。Leptin在无体重变化SCLC组较健康人群以及炎症患者增高，诊断的敏感度为50.11%，特异度86.77%；而Leptin在体重下降组较对照组无明显差异。此外，血清MSP-α、MIP-1β水平在三组之间比较无明显差异。

**结论:**

血清uPAR升高在SCLC中具有一定的诊断价值，而Leptin在无体重变化的SCLC中可能具有诊断意义。

## Abstract (en)

**Background and objective:**

It has been proven that serum cytokines could be changed in the early stage of patients with small cell lung cancer (SCLC). The current research investigates the different changes of cytockines and searches for potential biomarkers to improve the situation of the early diagnosis of SCLC.

**Methods:**

The differential expression cytokines were detected using Reybiotech G6/G7 analysis of microarrays in the serum of 4 SCLC patients, 4 normal controls, and 4 phlegmonosis controls. The differential cytokines were confirmed by enzyme-linked immunosorbentassay (ELISA) in 197 SCLC patients, 180 normal controls, and 97 phlegmonosis controls.

**Results:**

The levels of the 4 most valuable biomarkers in SCLC, including Leptin, MSP-α, uPAR, and MIP-1β, were significantly higher than that in the other groups with microaaray screening (*P* < 0.05). The ELISA results showed that the uPAR are much higher in SCLC patients than that in the controls, in which the diagnostic sensitivity and specificity were 52.93% and 83.36%, respectively. The Leptin level was significantly higher in SCLC patients who do not have obvious body weight lost, whereas no difference were found in the SCLC who have obvious body weight lost compared with control groups. The diagnostic sensitivity and specificity of Leptin are 50.11% and 86.77%. The MSP-α and MIP-1β level have no significant difference among the three groups.

**Conclusion:**

The uPAR elevated level is significant in indicating SCLC diagnoses. The Leptin may be associated with SCLC in patients who do not have a change in weight.

肺癌为最常见的恶性肿瘤，其中小细胞肺癌（small cell lung cancer, SCLC）占肺癌发病率的20%。SCLC患者初始治疗效果较好，但肿瘤容易复发、转移，治疗效果并不理想，其总体5年生存率 < 5%。目前针对SCLC的血清学诊断应用最广泛的肿瘤标志物包括神经元特异性烯醇化酶（neuron-specific enolase, NSE）和胃泌素释放肽前体（progastrin releasing peptide, ProGrp）等。肿瘤患者免疫防御损伤及免疫功能在疾病的早期已经开始发生变化，已发现很多细胞因子的表达都与肿瘤的发生发展有关。如：SCLC患者外周血和胸水IL-2水平下降，可能与肺癌的发生有关。Alison^[1]^的研究发现，吸烟人群血清IL-1β、IL-6、TNF-α、IL-4和IL-8、IL-7、IL-15水平增高，提示发生肺癌的可能性大。Yee^[2]^研究发现，血清结缔组织活性肽（CTAPⅢ）联合嗜中性细胞激活肽2（NAP-2）可作为临床前肺癌的检测指标。因此，为了全面系统地研究细胞因子在SCLC患者血清中的变化特点，我们应用细胞因子芯片初步检测了SCLC患者、良性疾病患者与健康人群血清中120种细胞因子，进一步应用酶联免疫吸附法（ELISA试剂盒）进行验证，旨在发现差异表达细胞因子，并探讨其在SCLC中的诊断价值。

## 1 材料与方法

### 1.1 研究病例

本研究选择第三军医大学大坪医院血清库2007年-2010年SCLC患者血清197例，其中，男性162例，女性35例，年龄40岁-72岁，中位年龄46岁；其中体重正常组132例，体重明显下降组65例（体重下降的评估标准为在肿瘤被确诊前6个月内体重下降≥5%的患者）。正常对照组为正常体检人员180例，其中男性130例，女性50例，年龄38岁-65岁，中位年龄49岁。良性疾病组为肺炎性疾病患者97例，其中男性55例，女42例，年龄21岁-52岁，中位年龄43岁。所有SCLC病例均有病理组织学诊断，其中局限期56例，广泛期141例。入选病例未伴发感染以及神经内分泌、高血压等方面的疾病，无自身免疫异常，无过敏、哮喘等病史。所有血清标本均为空腹静脉采血，离心分离并于-20 ℃保存备用。

### 1.2 细胞因子芯片检测及分析

各组选取4例样本使用Raybiotech G6/G7细胞因子抗体芯片（芯片包括IL、TGF、TNF、VEGF四个家族共120种细胞因子，购自Raybiotech，美国）进行检测，其中局限期及广泛期SCLC各2例均为未接受治疗的患者。芯片检测结果，每种细胞因子取3个发光点均值，使用IC归一化后的数据进行Cluster分析。差异蛋白筛选标准：q-value(%)≤5，同时差异倍数即Fold Change控制在1.5倍以上，样本数≥3。Ratio=病变/正常。

### 1.3 ELISA检测

人瘦素（Leptin）、巨噬细胞刺激蛋白（macrophage stimulating protein α, MSP-α）、尿激酶型纤溶酶原激活剂受体（urokinase plasminogen activator receptor, uPAR）、巨噬细胞炎症蛋白1β（macrophage inflammatory protein 1β, MIP-1β）ELISA检测试剂盒购自R & D公司（美国），包括预包被96孔酶标板、洗涤缓冲液、终止液、稀释液、冻干标准品一组、底物缓冲液、ABTS使用液及TMB（四甲基联苯胺）使用液，ELISA法按照试剂盒操作说明书的步骤进行。

### 1.4 统计学处理

细胞因子抗体芯片检测的原始数据进行SAM方差分析后将所有*P*＜0.05的细胞因子进行Cluster 3.0聚类分析，从而筛选出SCLC组相对于正常对照和炎症组的差异表达细胞因子。ELISA检测数据采用SPSS 13.0统计软件进行单因素方差分析。检测指标敏感度=真阳性例数/（真阳性例数+假阴性例数）；特异度=真阴性例数/（真阴性例数+假阳性例数）；准确性=（真阳性例数+真阴性例数）/（真阳性例数+假阴性例数+真阴性例数+假阳性例数）。

## 2 结果

### 2.1 细胞因子芯片检测结果

使用Raybiotech G6/G7120位点细胞因子抗体芯片进行检测，聚类图显示SCLC、炎症和正常样本被明显区分开（图 1）。在120种细胞因子中，SCLC与正常组的差异表达细胞因子65种，SCLC和炎症组与正常组的差异表达细胞因子60种，炎症组与正常组的差异表达细胞因子55种，而SCLC与炎症组的差异表达细胞因子仅见Leptin。根据差异表达细胞因子组间比较结果（表 1），我们对Leptin、MSP-α、uPAR、MIP-1β四种细胞因子进行进一步验证。

**1:**
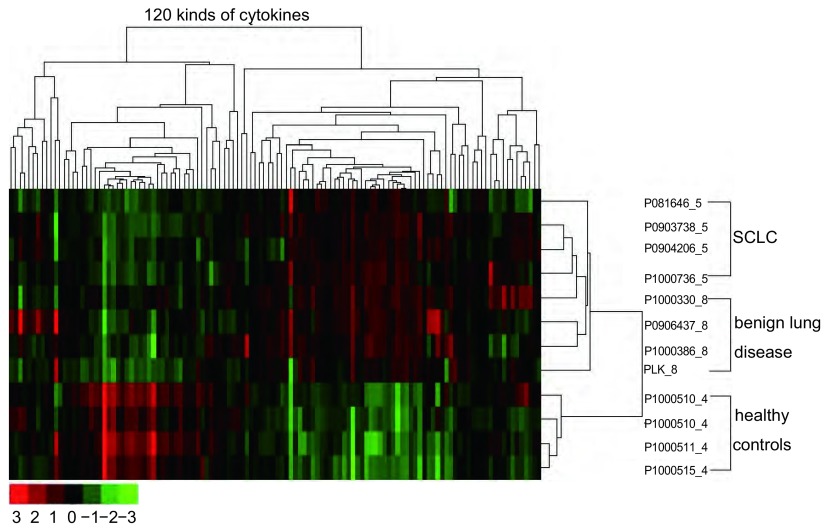
Raybiotech G6/G7芯片检测结果。疾病及两个对照组血清样本共12个。首先对样本蛋白浓度的结果使用SAM分析方法，然后进行聚类分析。聚类图显示SCLC，炎症和正常样本明显分离。 The results of Raybiotech G6/G7 Microarrays detection. A total of 12 serum samples were separated into a disease set and two control sets. The results of signaling protein concentrations were analyzed with SAM method initially, and this was followed by cluster analysis. Cluster diagram shows the SCLC, inflammation and normal samples are significantly separated. SCLC: small cell lung cancer.

**1:** 四种差异表达细胞因子组间比较结果 Comparison of expression level of the differential expression cell factors in the different groups

Biomarker	Comparison between groups	Score (d)	Numerator (r)	Denominator (s+s0)	Fold change	q-value (%)
MSP-*α*	Disease/healthy^*^	2.904	2.750	0.947	9.953	0
	Inflammation/healthy^*^	1.806	1.980	1.097	6.961	1.726
	Inflammation/SCLC	-1.298	-1.539	1.186	0.538	100
	SCLC/healthy^*^	6.589	3.520	0.534	12.944	0
MIP-1*β*	Disease/healthy^*^	2.464	1.790	0.726	3.138	0
	Inflammation/healthy^*^	2.358	2.176	0.923	4.230	0.895
	Inflammation/SCLC	1.052	0.773	0.735	2.067	41.25
	SCLC/healthy^*^	1.835	1.403	0.765	2.046	0.679
uPAR	Disease/healthy^*^	2.216	1.434	0.647	3.187	0
	Inflammation/healthy^*^	5.228	2.036	0.389	4.245	0
	Inflammation/SCLC	1.661	1.204	0.725	1.993	26.190
	SCLC/healthy^*^	1.180	0.832	0.705	2.130	4.212
Leptin	Disease/healthy	-0.449	-0.574	1.277	0.936	21.290
	Inflammation/healthy	0.540	0.763	1.411	1.700	23.889
	Inflammation/SCLC^*^	2.365	2.673	1.130	9.865	0
	SCLC/healthy	-1.771	-1.911	1.079	0.172	1.718
MSP-*α*: macrophage stimulating protein *α*; uPAR: urokinase plasminogen activator receptor; MIP-1*β*: macrophage inflammatory protein 1*β*.						

**2:**
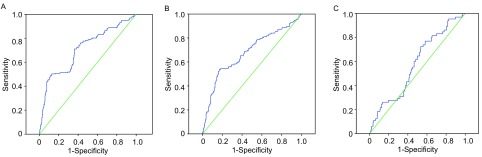
ROC曲线分析结果。A：uPAR。曲线下面积0.709，*P*＜0.05，在SCLC组和对照组间有统计学差异。B：Leptin（normal weight）。曲线下面积0.679，*P*＜0.05，在SCLC组和对照组间有统计学差异。C：Leptin（weight lost）。曲线下面积0.577，*P*=0.053＞0.05，在SCLC组和对照组间没有统计学差异。 he results of ROC curve analysis. A: uPAR. Area under the curve 0.679, *P* < 0.05, SCLC group verse control group: statistical significance. B: Leptin (normal weight). Area under the curve 0.679, *P* < 0.05, SCLC group verse control group: statistical significance. C: Leptin (weight lost). Area under the curve 0.577, *P*=0.053 > 0.05, SCLC group verse control group: no statistical significance.

### 2.2 ELISA检测结果

197例SCLC患者血清uPAR为（2.891, 8±1.831, 08）ng/L，较健康人群（1.338, 1±0.283, 79）ng/L以及炎症患者（1.314, 4±0.3053, 6）ng/L明显升高（*P*＜0.05）。无明显体重变化的132例SCLC患者血清Leptin为（4.333, 1±2.786, 69）ng/L，较健康人群（1.819, 6±0.436, 13）ng/L以及炎症患者（1.660, 4±0.398, 03）ng/L明显升高，其差异有统计学意义（*P*＜0.05）。而体重下降的65例SCLC患者血清Leptin较对照组无明显差异。此外，血清MSP-α、MIP-1β水平在三组之间无明显差异。ROC曲线分析结果显示：uPAR在SCLC组和对照组间有统计学差异（*P*＜0.05），Leptin在体重正常的SCLC组和对照组间有统计学差异（*P*＜0.05），Leptin体重下降后的SCLC组和对照组间没有统计学差异（*P*＞0.05）。血清uPAR诊断SCLC的敏感度及特异度分别为50.11%和86.77%。体重无明显变化的SCLC患者血清Leptin诊断SCLC的敏感度及特异度分别为83.36%和52.93%，二者联合诊断SCLC的敏感度及特异度分别为50.37%和92.68%（表 2）。

**2:** 检测的敏感度及特异度分析 Analysis of the sensitivity and specificity in detection

Biomarker	SCLC number	Controls	Sensitivity	Specificity	Accuracy
Leptin	132 (normal weight)		52.93%	83.36%	60.52%
Leptin	65 (weight lost)	BLD: 97	26.60%	87.32%	42.51%
uPAR	197	HC: 180	50.11%	86.77%	59.33%
Leptin+uPAR	197		50.37%	92.68%	71.62%
BLD: benign lung diseases; HC: healthy controls.					

## 3 讨论

细胞因子是由淋巴细胞、成纤维细胞、单核巨噬细胞等产生的一种分泌型糖蛋白。细胞因子之间可相互调节并形成复杂的调控网络，它们是免疫系统的信息传递介质，也是神经、内分泌及其它系统和免疫系统相互联系的桥梁，在如炎性疾病进展、恶性肿瘤的发生、进展和复发过程中也起着一定的作用^[3]^。目前，细胞因子在肺癌的研究多针对非小细胞肺癌，而关于小细胞肺癌尚缺乏系统的研究。此外，传统的细胞因子检测方法相对落后，细胞因子抗体芯片技术应用类似酶联免疫反应的原理，将特异性细胞因子抗体按预定的阵列形式和顺序排列在载体芯片上，将待测的标本与之反应，对芯片进行扫描，同时计算机软件对荧光信号进行分析，即可获得准确的结果。该技术还具有高通量、高特异度、高灵敏度等特点。

肿瘤的发生发展与炎症密切相关，而细胞因子IL家族在肿瘤相关炎症中具有重要作用，此外还包括MMP家族、TGF家族、TNF家族、VEGF家族。本研究应用的Raybiotech G6/G7抗体芯片含有120种细胞因子，包括了IL、TGF、TNF、VEGF家族的多数蛋白。芯片检测结果显示，疾病（SCLC和炎症）与正常样本有明显差异，聚类分析可以将三者明显分开，表明SCLC与炎症及正常对照人群的细胞因子表达谱是存在差异的。应用芯片进行筛选我们也证实了许多已报道的小细胞癌相关细胞因子有统计学差异，本研究在SCLC组与健康组的比较中，同时表达增高的有56种，包括IL-4、IL-2、TNFβ、TNFα、IGF-Ⅰ等与肿瘤发生发展密切相关的细胞因子。因为炎症反应对机体细胞因子整体水平影响较大，因此需要同时对比SCLC组相对于炎症组表达增高的指标。由于本研究的目的是希望找到SCLC患者血清中较为特异细胞因子，故仅对uPAR、Leptin、MSP-α、MIP-1β进行了进一步的验证。结果显示SCLC患者血清uPAR较健康人群以及炎症患者增高，Leptin在无体重变化SCLC组较健康人群以及炎症患者增高，而Leptin在体重下降组较对照组无明显差异。此外，血清MSP-α、MIP-1β水平在三组之间无明显差异。

纤溶酶原激活系统与肿瘤的转移侵润有明显关系，肿瘤细胞最初产生无生物活性的uPA酶原与肿瘤细胞表面的特定受体结合后，被纤溶酶激活，激活的uPA催化纤溶酶原生成纤溶酶，形成正反馈效应，破坏细胞外基质和细胞基膜，因此，尿激酶型纤溶酶原激活剂和纤溶酶原激活物抑制剂与肿瘤浸润密切相关^[4]^。同时，一些研究^[5, 6]^证实许多肿瘤原发病灶和转移部位的细胞和其周围临近细胞uPA及其受体的表达水平增高。本研究ELISA验证结果显示SCLC患者血清尿激酶型纤溶酶原激活剂受体较健康人群以及炎症患者明显升高，进一步证实uPAR可能是诊断SCLC有价值的血清标志物。

Leptin具有167个氨基酸序列的16 kDa的蛋白质，定位于7号染色体的*ob*基因，主要为白色脂肪组织产生，胎盘、胃基底细胞以及胰腺可产生少量的Leptin。其ob启动子可被很多转录因子所诱导包括HIF-1^[7]^、过氧化物酶体活性受体激动剂^[8]^等。研究表明Leptin在子宫内膜癌、胃癌等组织中的表达明显高于癌旁组织，提示Leptin可能具有影响肿瘤细胞分化、增殖的能力^[9]^，Somasundar^[10]^进一步证实了Leptin是一种新的促肿瘤生长因子，可刺激肿瘤新生血管的形成，因此在肺癌的发生和进展中发挥着重要的作用。Ribeiro等^[11]^的研究也表明Leptin基因启动子区的功能多态性增加了3倍患非小细胞肺癌的风险，其过表达增加了肺癌发生的可能性。

Leptin是一种调节脂肪含量且增加机体能量消耗的细胞因子，动物实验证实Leptin具有促进肺组织成熟，加强通气功能的作用，并证明BMI指数增高可诱发小鼠死于肿瘤^[12]^，同时确定肥胖增加了肺癌的易感性^[13, 14]^。大量的研究表明Leptin在肿瘤引起的体重丢失及肿瘤恶病质中可能具有重要作用，但结果并不一致。Carpagnan等^[15]^提出在NSCLC患者中血清Leptin水平相对于对照组明显增高，然而Karapanagiotou等^[16]^报道了在进展期NSCLC中，血清Leptin水平与患者性别和体重无关，其表达程度与肺癌的组织学类型、分化程度、分期、总生存率以及患者是否发生恶病质无关，提示Leptin不能作为NSCLC诊断和预后的参考指标。本研究结果显示体重无变化的SCLC患者在未接受临床治疗前的血清Leptin水平较对照组有明显差异，而体重下降的患者其Leptin水平与对照组无明显变化。该结果提示Leptin水平升高与SCLC患者体重下降没有明显关系，甚至可能是相反的作用，而对体重无变化的SCLC患者血清Leptin水平升高可能具有一定的诊断价值。

巨噬细胞刺激蛋白-α又称为肝细胞样生长因子^[17]^，可诱导巨噬细胞激活，并促进巨噬细胞趋化作用^[18]^，同时可抑制巨噬细胞中NO合酶mRNA（信使核糖核酸）的表达^[19]^等。巨噬细胞炎症蛋白1β是趋化因子受体CCR1的配体，可由多种细胞产生。研究发现其表达水平与肿瘤的淋巴结转移有关，提示MIP可能参与了肿瘤的转移^[20, 21]^，同时MIP通过抑制树突状细胞的成熟达到降低其对肿瘤的局部免疫反应^[22, 23]^。目前针对MSP-α及MIP-1β在肺癌中的研究较少，大多为探讨二者在细胞因子网络中与其他细胞因子的相互作用。本文细胞因子芯片检测结果显示，除炎症组与SCLC组进行组间比较无意义之外，其余各组比较皆有统计学差异，提示炎症的发生对MSP-α及MIP-1β水平影响较大。然而，ELISA检测并没有证实其在芯片检测中相同的结果，因此，对二者在SCLC中的作用还有待进一步研究。

目前诊断SCLC的敏感血清肿瘤标志物包括神经元特异性烯醇化酶（敏感度39.73%、特异度89.11%）、胃泌素释放肽前体（ProGrp）（敏感度51.48%、特异度94.89%）^[24]^和DKK1（Dickkopf-1, DKK1）^[25]^等。本组结果血清uPAR诊断SCLC的敏感度及特异度分别为50.11%和86.77%，体重无明显变化的SCLC患者血清Leptin诊断的敏感度及特异度分别为83.36%和52.93%，而二者联合诊断的特异度可提高到92.68%，表明uPAR及Leptin联合检测在SCLC诊断中可能更具有临床价值。
